# EXPERIENCES EMBEDDING MIND AT HOME DEMENTIA CARE COORDINATION PROGRAM IN PRIMARY CARE

**DOI:** 10.1093/geroni/igae098.1346

**Published:** 2024-12-31

**Authors:** Elizabeth Ciemins, Quincy Samus, Stephen Shields, Mia Yang, Shelby Herrig, Rachel Breyfogle, Alicia Rooney, Monette McKinnon

**Affiliations:** American Medical Group Association, Alexandria, Virginia, United States; Johns Hopkins University, Baltimore, Maryland, United States; American Medical Group Association, Alexandria, Virginia, United States; Wake Forest University School of Medicine, Winston-Salem, North Carolina, United States; McFarland Clinic, Ames, Iowa, United States; McFarland Clinic, Ames, Iowa, United States; American Medical Group Association, Alexandria, Virginia, United States; American Medical Group Association, Alexandria, Virginia, United States

## Abstract

The prevalence of people living with dementia (PLWD) is growing and dementia is one of the highest burden, and highest cost chronic conditions in the US. Care management support within primary care practices is one solution to provide patient- and care partner (CP)-centered care, improve patient care outcomes, e.g., hospitalizations, ED visits, polypharmacy, high care partner strain, and reduce healthcare costs. This session describes a collaborative adaptation and implementation of the MIND at Home Dementia Care Coordination Program into two large primary care clinics in two states (e.g., Iowa and North Carolina). The program pilot expanded the skills of existing primary care staff members to the level of Memory Care Coordinators (MCCs), who worked with larger primary care teams on combining the benefits of clinic-based with home-based services that support PLWD, their families, and CPs. 106 primary care patients and their CPs were recruited for a 3-month intervention period and process measures, including identified and addressed needs of the PLWD and CP, were ascertained. This session will discuss the feasibility of program implementation in a racially, economically, and geographically diverse population of PLWD and provide qualitative and quantitative outcomes on patient, provider and MCC program acceptability, program impact, and barriers and facilitators to implementation from the primary care practice perspective. The presentation will reflect on specific issues important to consider in primary care including underdiagnosis of dementia, patient recruitment and outreach, incorporation of home visits, interdisciplinary collaboration and consultation, and outcomes ascertainment.

